# Littoral Cell Angioma of the Spleen Presenting With Hypersplenism: A Case Report

**DOI:** 10.7759/cureus.116721

**Published:** 2026-09-22

**Authors:** Salma A Tohfa, Mahamud Riyad, Muntasir Faisel, Murshidul Arefin, Marjan Jamila

**Affiliations:** 1 Surgery, Popular Medical College and Hospital, Dhaka, BGD; 2 Department of Hepatobiliary, Pancreatic and Liver Transplant Surgery, Bangabandhu Sheikh Mujib Medical University (BSMMU), Dhaka, BGD; 3 Surgery, Sir Salimullah Medical College, Dhaka, BGD

**Keywords:** case report, hypersplenism, littoral cell angioma, spleen, splenectomy, vascular tumor

## Abstract

Littoral cell angioma (LCA) is a rare primary vascular neoplasm of the spleen that may present with nonspecific symptoms, diagnostic dilemmas, and haematological abnormalities related to hypersplenism. We report the case of a 50-year-old man who presented with a three-month history of generalised weakness, intermittent fever, anorexia, and progressive abdominal fullness. Physical examination revealed marked splenomegaly extending to the umbilicus. Laboratory investigations demonstrated anaemia, thrombocytopenia, and leucopenia, while peripheral blood film showed microcytic hypochromic anaemia with thrombocytopenia. Bone marrow examination, haemoglobin electrophoresis, iron profile, and liver function tests revealed no significant abnormalities.

Abdominal ultrasonography demonstrated marked splenomegaly, with the spleen measuring about 15.5 x 6.5 cm and multiple ill-defined hypoechoic space-occupying lesions within it, the largest measuring about 3.4 x 2.5 cm. Following multidisciplinary evaluation, the patient underwent laparoscopic splenectomy for symptomatic splenomegaly and hypersplenism. Histopathological examination demonstrated multiple anastomosing, blood-filled vascular channels lined by tall endothelial cells with focal haemophagocytosis, consistent with LCA. The postoperative course was uneventful, and the patient was discharged on the sixth postoperative day with improvement in his presenting symptoms and haematological abnormalities.

LCA is an uncommon vascular tumour with a variable clinical presentation ranging from incidental detection to symptomatic hypersplenism. Imaging findings are nonspecific. Splenectomy remains both a diagnostic and a therapeutic modality. LCA should be considered in the differential diagnosis of splenomegaly with multiple splenic lesions and pancytopenia. Histopathological examination following splenectomy is essential for definitive diagnosis.

## Introduction

Primary splenic tumours are rare pathological entities that may be broadly classified as benign or malignant. Primary vascular tumours of the spleen include haemangioma, hamartoma, lymphangioma, haemangioendothelioma, littoral cell angioma (LCA), and angiosarcoma, with malignant vascular tumours being considerably less common [1]. LCA is an exceptionally rare primary vascular neoplasm of the spleen that arises from the littoral cells lining the venous sinuses of the splenic red pulp. These specialised cells show both endothelial and histiocytic differentiation and are involved in the sequestration and phagocytosis of senescent erythrocytes [2,3]. LCA was first described by Falk et al. in 1991 as a distinctive splenic vascular lesion with histiocytic differentiation [2].

The clinical presentation of LCA is variable. Many patients remain asymptomatic, and the lesions are detected incidentally during abdominal imaging. Symptomatic patients may present with splenomegaly, abdominal discomfort, fever, anaemia, thrombocytopenia, or other manifestations of hypersplenism [3,4]. Radiological findings are nonspecific, and LCA may present as multiple hypoattenuating splenic lesions in an enlarged spleen, which makes differentiation from other benign and malignant splenic lesions difficult [5]. Definitive diagnosis therefore depends on histopathological examination and immunohistochemical evaluation.

LCA has also been reported in association with visceral malignancies and immune-mediated disorders. Peckova et al. reported a frequent association between LCA and visceral malignancies in a series of 25 patients, suggesting that patients with this diagnosis may benefit from appropriate clinical evaluation for associated disease [6]. We report a rare case of LCA of the spleen in a 50-year-old man who presented with symptomatic splenomegaly, generalised weakness, intermittent fever, anorexia, and abdominal fullness. The patient successfully underwent laparoscopic splenectomy, with histopathological confirmation of LCA and subsequent improvement in his presenting symptoms and haematological abnormalities.

## Case presentation

A 50-year-old man presented with generalised weakness, intermittent fever, anorexia, and progressive abdominal fullness for three months. He had multiple prior hospital admissions for anaemia and fever. There was no history of abdominal trauma, altered bowel habits, or significant weight loss. His medical history was significant for type 2 diabetes mellitus managed with gliclazide, with no history of other comorbidities, immune disorders, or malignancy. He had no prior history of surgery. He denied smoking or alcohol consumption. On physical examination, massive splenomegaly, measuring approximately 16 cm, was noted, extending up to the umbilicus. No signs of peritoneal irritation, hepatomegaly, or lymphadenopathy were observed.

Laboratory investigations revealed anaemia, thrombocytopenia, and leukopenia consistent with hypersplenism. Peripheral blood film demonstrated microcytic hypochromic anaemia with leukopenia and thrombocytopenia. Bone marrow examination, haemoglobin electrophoresis, iron profile, and liver function tests were within normal limits (Table 1).

**Table 1:** Laboratory investigations

Investigation	Patient value	Unit	Reference range
Haemoglobin	6.9	g/dL	13.0–17.0
White blood cell count	3.1	×10⁹/L	4.0–10.0
Platelet count	55	×10⁹/L	150–410
Mean corpuscular volume	68	fL	81–94
Peripheral blood film	Microcytic hypochromic anemia with leucopenia with thrombocytopenia	—	—
Bone marrow examination	No significant abnormality	—	—
Haemoglobin A	97.3	%	96.8–97.8
Haemoglobin A₂	2.7	%	2.2–3.2
Serum iron	60	µg/dL	49–181
Total iron-binding capacity	296	µg/dL	291–430
Ferritin	92.05	ng/mL	20–300
Liver function tests	Within normal limits	—	—

Ultrasonography of the abdomen demonstrated an enlarged spleen, measuring approximately 15.5 × 6.5 cm, with multiple ill-defined hypoechoic areas, the largest measuring approximately 3.4 × 2.5 cm, suggestive of marked splenomegaly with multiple space-occupying lesions within the spleen (Figures 1-2). In view of the progressive splenomegaly and cytopenia, the patient was discussed in a multidisciplinary team meeting, and laparoscopic splenectomy was planned following appropriate vaccination administered two weeks before the operation.

**Figure 1:**
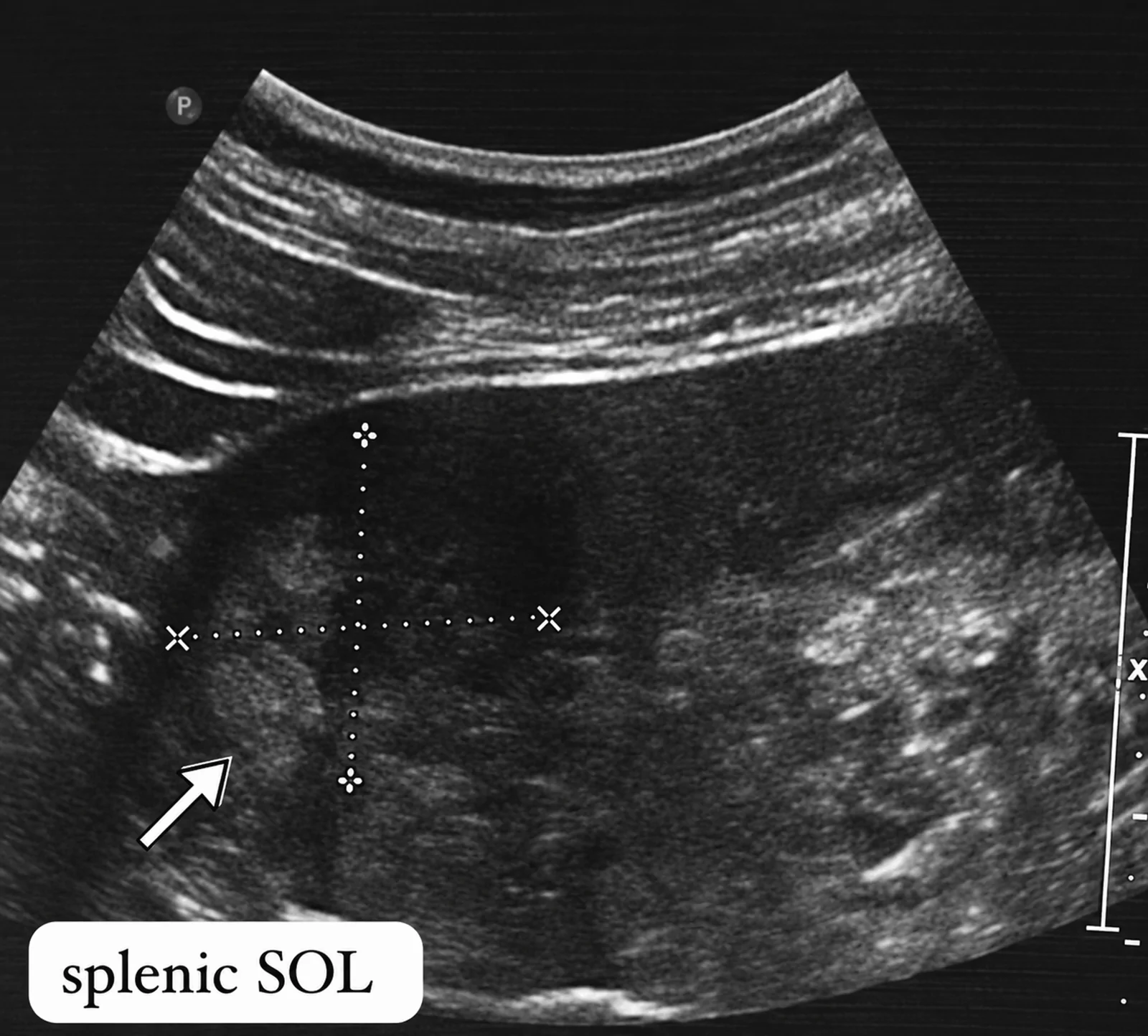
Ultrasonography showing splenic SOL SOL: space-occupying lesion

**Figure 2:**
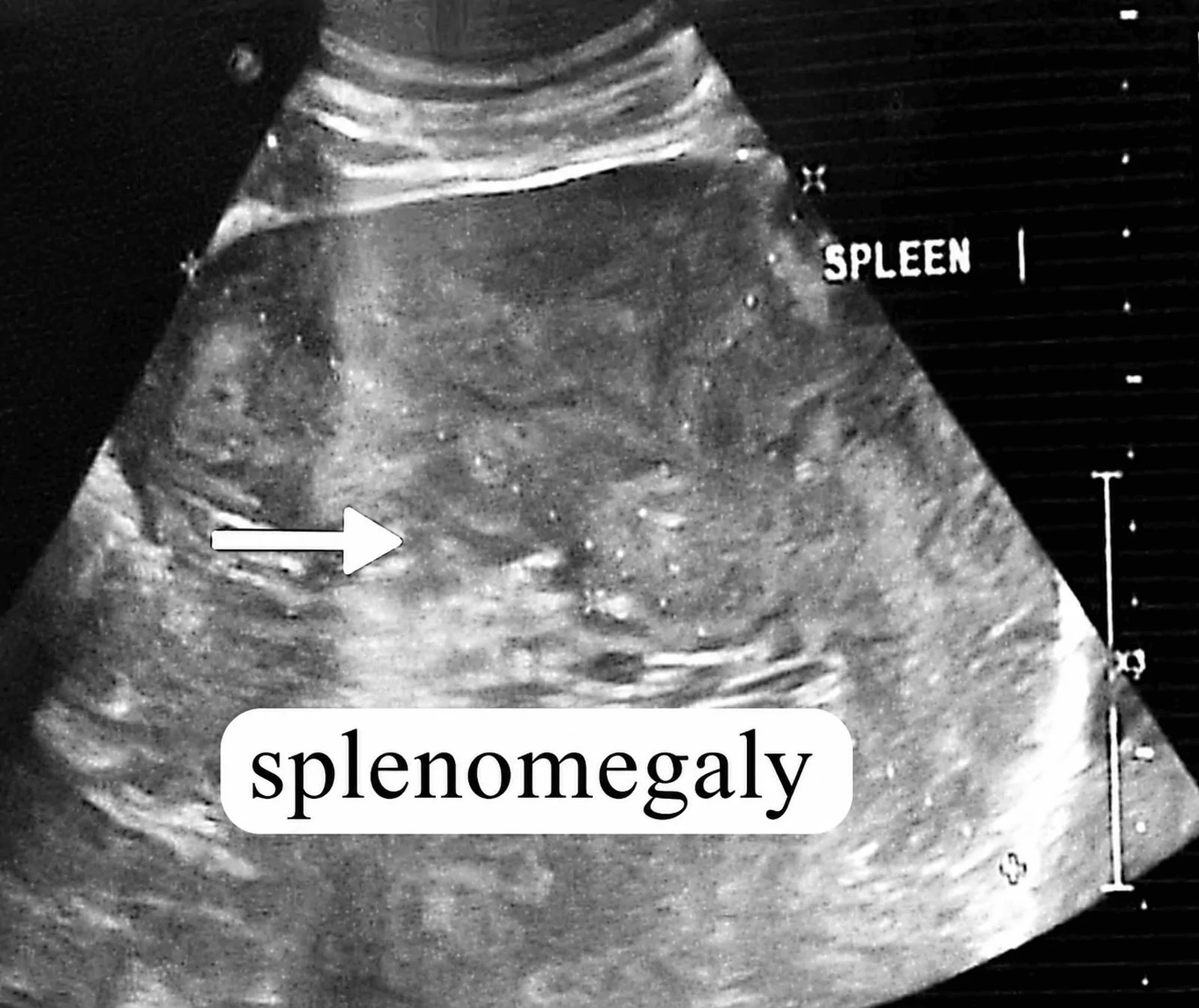
Ultrasonography showing marked splenomegaly

Laparoscopic splenectomy was successfully performed, and the specimen was sent for histopathological evaluation (Figures 3-4). Histopathological examination demonstrated multiple anastomosing blood-filled vascular channels lined by tall endothelial cells with focal haemophagocytosis, findings consistent with LCA of the spleen. Immunohistochemistry was not done. 

**Figure 3:**
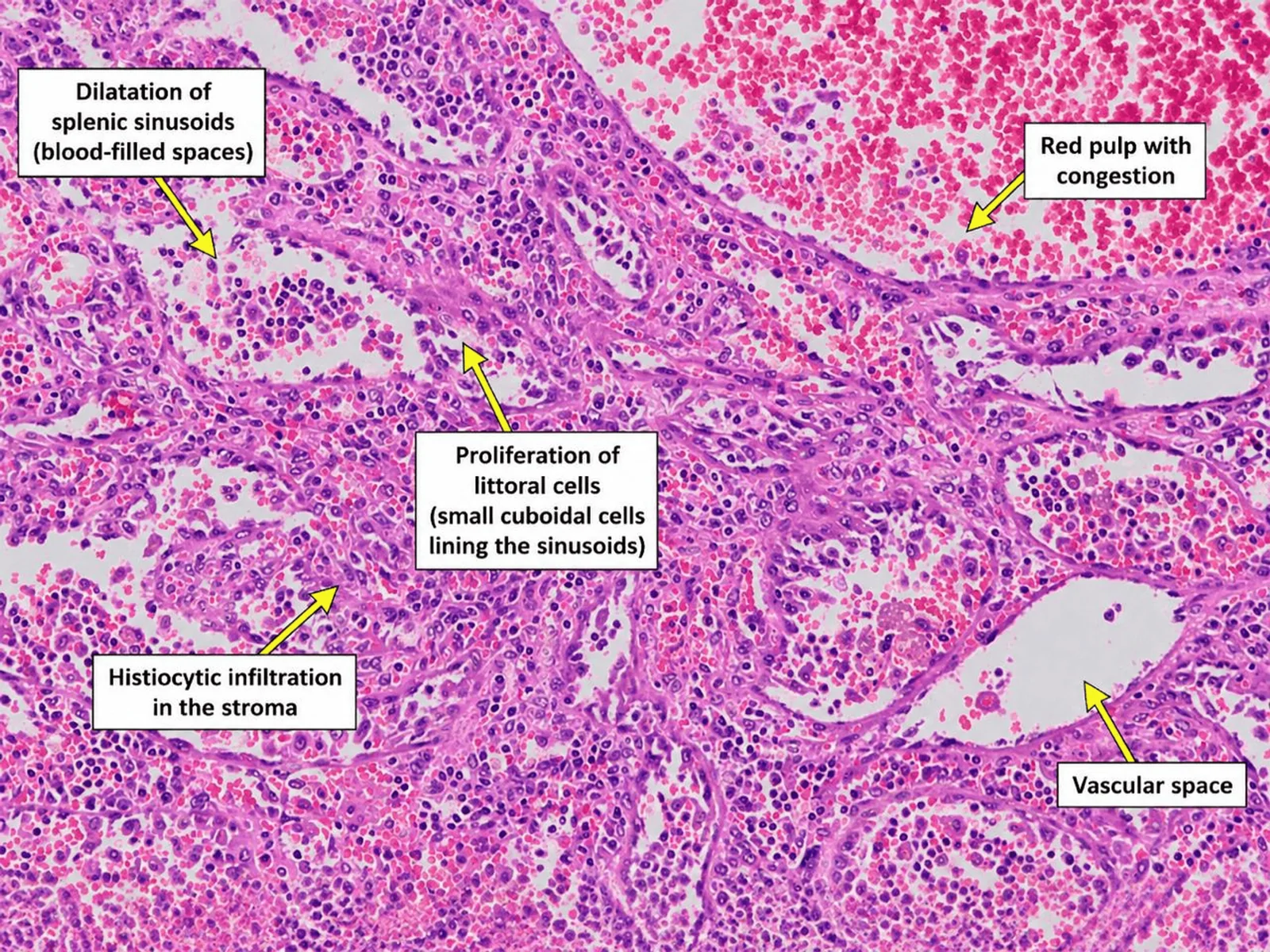
Histopathology slide - image 1 The slide shows proliferation of anastomosing, tortuous, blood-filled vascular channels with proliferation of littoral cells, histiocytic infiltration in the stroma, and dilatation of splenic sinusoids. The white pulp is unremarkable

**Figure 4:**
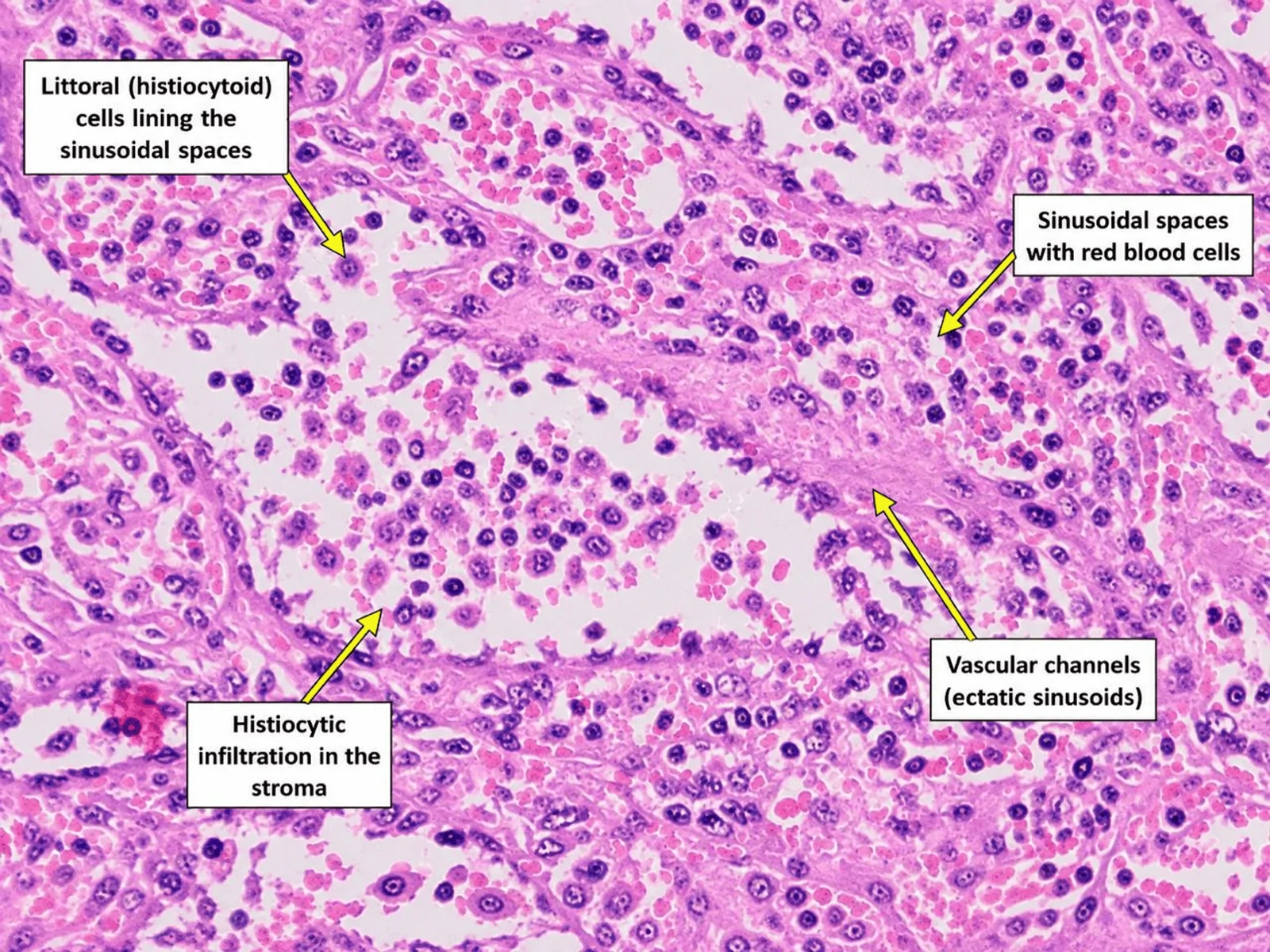
Histopathology slide - image 2 The histopathology slide shows proliferation of anastomosing, tortuous, blood-filled vascular channels and littoral cells lining the sinusoidal spaces. The white pulp is unremarkable

The postoperative recovery was uneventful, and the patient was discharged on postoperative day six. At the 14th-day postoperative follow-up, the patient’s platelet count had improved to 259 ×10⁹/L, while the haemoglobin level increased to 11.3 g/dL. A mild leukocytosis was noted, with a white blood cell count of 11.51 ×10⁹/L. The patient was clinically well with improvement in his presenting symptoms.

## Discussion

LCA is a rare primary vascular neoplasm of the spleen originating from the littoral cells of the splenic red pulp. These cells demonstrate both endothelial and histiocytic differentiation, which accounts for the characteristic pathological and immunophenotypic features of the tumour [2,3]. The clinical presentation of LCA is variable. Many cases are incidentally detected, whereas symptomatic patients may present with splenomegaly, anaemia, thrombocytopenia, fever, abdominal discomfort, or other manifestations of hypersplenism [3,4]. Our patient presented with generalised weakness, intermittent fever, anorexia, and progressive abdominal fullness associated with marked splenomegaly and cytopenias. Bone marrow examination showed no significant abnormality, supporting a peripheral cause for the cytopenias. The subsequent improvement of the haematological abnormalities following splenectomy further supports hypersplenism as an important clinical manifestation in this patient.

Radiological diagnosis of LCA remains challenging because its imaging appearance is nonspecific. CT commonly demonstrates multiple hypoattenuating lesions within an enlarged spleen. In a clinicopathological and CT study of eight patients, all patients demonstrated laboratory evidence of hypersplenism, and most had splenomegaly with multiple hypoattenuating splenic masses [5]. Our patient similarly demonstrated marked splenomegaly with multiple space-occupying lesions on ultrasonography. The differential diagnosis of multiple splenic lesions includes haemangioma, hamartoma, lymphoma, metastases, and other vascular neoplasms.

Histopathology remains essential for definitive diagnosis. LCA is characterised by anastomosing vascular channels and cyst-like spaces lined by distinctive tall endothelial cells, often containing blood and demonstrating haemophagocytosis [2,3]. The histological findings in our patient were consistent with these characteristic features. Immunohistochemistry is particularly useful because LCA demonstrates combined endothelial and histiocytic differentiation. Reported cases have shown expression of endothelial markers such as CD31 together with histiocytic markers such as CD68, while CD34 may be negative or only focally expressed [3,7]. In the present case, the diagnosis was established on histopathological examination, as the patient declined immunohistochemistry.

The differential diagnosis includes splenic haemangioma, hamartoma, lymphoma, and angiosarcoma. Angiosarcoma is particularly important to exclude because it is an aggressive malignant vascular neoplasm. Histological assessment helps distinguish LCA from angiosarcoma based on the degree of cytological atypia, mitotic activity, and destructive growth pattern [1,3]. The aetiology of LCA remains uncertain. An association with immune dysregulation has been proposed because of its reported coexistence with autoimmune and inflammatory conditions. In addition, LCA has been associated with visceral malignancies. Peckova et al. studied 25 cases and reported a frequent association with visceral malignancies, emphasising the importance of appropriate clinical assessment after diagnosis [6]. However, these associations do not establish a causal relationship.

Management depends on the clinical presentation and extent of splenic involvement. In symptomatic patients with splenomegaly, hypersplenism, or lesions in which malignancy cannot be excluded, splenectomy provides both diagnostic confirmation and definitive treatment. Laparoscopic splenectomy has been successfully reported for LCA, and comparative experience has demonstrated its feasibility in appropriately selected patients [8,9]. In our patient, the combination of symptomatic splenomegaly, multiple splenic lesions, and pancytopenias supported surgical management. Laparoscopic splenectomy was completed uneventfully, followed by improvement in the patient’s symptoms and haematological abnormalities.

Although LCA is generally regarded as a benign lesion, malignant variants and littoral cell angiosarcoma have been reported. Therefore, histopathological assessment is important not only for confirming LCA but also for excluding malignant vascular neoplasms. Appropriate postoperative clinical evaluation for associated malignancy and immune-mediated disease may also be considered, particularly in patients with relevant clinical features [6].

This case report highlights the importance of considering LCA in patients with splenomegaly, multiple splenic lesions, and unexplained pancytopenias. Its nonspecific clinical and radiological presentation can make preoperative diagnosis difficult, while histopathological examination provides the definitive diagnosis.

## Conclusions

LCA is a rare primary vascular tumour of the spleen that should be considered in the differential diagnosis of patients presenting with splenomegaly and unexplained haematological abnormalities. Owing to its nonspecific clinical and radiological presentation, definitive diagnosis relies on histopathological evaluation following splenectomy. The present case also demonstrates that laparoscopic splenectomy can be a safe and effective diagnostic and therapeutic approach in selected patients.
